# Epidemiology of carbapenem-resistant Enterobacterales infections in Tennessee, 2016–2022

**DOI:** 10.1017/S0950268825100514

**Published:** 2025-09-08

**Authors:** Daniel Muleta, Simonne S. Nouer, Elizabeth A. Tolley, Raquel M. Villegas, Jacquelyn Taylor, Melphine M. Harriott

**Affiliations:** 1 Division of Preventive Medicine, Department of Preventive Medicine, College of Medicine-Memphis, University of Tennessee Health Science Center, Memphis, TN, USA; 2 Healthcare-Associated Infections and Antimicrobial Resistance Program, Communicable and Environmental Diseases and Emergency Preparedness Division, Tennessee Department of Health, Nashville, TN, USA; 3 Tennessee Emerging Infections Program at Vanderbilt, Vanderbilt University Medical Center, Nashville, TN, USA

**Keywords:** Carbapenem resistant, Infection prevention, Surveillance, Trends, Carbapenemase production

## Abstract

This surveillance report describes the epidemiology and clinical outcomes of carbapenem-resistant Enterobacterales (CRE) infections in Tennessee from 2016 to 2022, analysing 570 cases and 406 isolates. The incidence of CRE infections per 100 000 population showed an upward trend. *Enterobacter* species were the most common organisms, whereas *Klebsiella* species were the main carbapenemase-producing CRE (CP-CRE). *Klebsiella pneumoniae* carbapenemase was the most common mechanism contributing to this resistance. Demographic characteristics of patients with identified isolates demonstrated a median age of 69.5 years. There were no significant differences in CP-CRE infection by sex or race. Patients with CP-CRE were more likely to be hospitalized than those with non-CP-CRE, at 60.9% and 43.9%, respectively. Multivariable analysis indicated that patients with CP-CRE had significantly higher odds of 90-day mortality (odds ratio, 2.22; 95% confidence interval, 1.12–4.42; *p* < 0.0001) than non-CP-CRE patients. Individuals with a higher Charlson Comorbidity Index score exhibited an increased odds of dying within 30- and 90-day post-specimen collection and had a greater likelihood of requiring intensive care unit admission. This report underscores the need to understand the epidemiology and risk factors linked to CRE infections to improve prevention strategies and patient care.

## Introduction

The emergence of multidrug-resistant organisms (MDROs) presents a pressing public health challenge, particularly in healthcare settings [1]. With an estimated 2.8 million infections and over 35 000 deaths annually attributed to MDROs, the situation is dire [1]. Among these, the increasing prevalence of carbapenem-resistant Enterobacterales (CRE) is of particular concern [1, 2]. Enterobacterales, established human pathogens, are associated with a variety of clinical infections, including urinary tract infections, gastrointestinal infections, wound infections, and bloodstream infections [3]. These organisms can acquire mobile genetic elements that encode carbapenemases, enzymes that render carbapenems ineffective [4]. CRE that produce carbapenemase are known as carbapenemase-producing CRE (CP-CRE). The implications of CRE infections on mortality, patient health outcomes, and healthcare costs are significant [5–7].

The Centers for Disease Control and Prevention (CDC) collaborates with various states through the Emerging Infections Program to conduct population- and laboratory-based surveillance of pathogens that pose significant public health threats. This partnership focuses on enhancing our understanding of the rapid dissemination and increasing prevalence of antimicrobial-resistant pathogens, including CRE. CRE surveillance constitutes a key component of the Multi-site Gram-negative Surveillance Initiative, in which the Tennessee Department of Health has been an active participant since 2014. This surveillance report aims to describe the epidemiology, risk factors, and clinical outcomes associated with infections caused by CRE and CP-CRE in Tennessee from 2016 to 2022. Our goal is to enhance the understanding of the prevalence and clinical implications of CRE and CP-CRE in the region. Ultimately, we hope to use these data to improve our infection prevention strategies, allowing us to implement targeted measures that reduce adverse health outcomes.

## Methods

### Study design

This was a retrospective, observational report of CRE in Tennessee from 2016 to 2022. The surveillance was conducted as part of the Emerging Infections Program, which was established by the CDC. The CDC developed the study protocol and a case definition. Once cases were confirmed to meet the specified criteria, medical records of each patient were reviewed, and a case report form was completed to collect data on risk factors, demography, laboratory testing results, comorbidities, and other risk factors. The Charlson Comorbidity Index (CCI) score was calculated for each case using the comorbidity information collected from the case report form. Mortality data were obtained from the Tennessee Department of Health Vital Records Death Registry. Laboratories participating in the surveillance study were required to send all isolates that met the case definition (see the ‘Case definition’ section below) for additional testing. However, not all isolates were submitted. Isolates that were submitted were tested at the Tennessee State Public Health Laboratory to assess carbapenemase production and identify genes mediating resistance. Carbapenem-resistant isolates were screened using the modified carbapenem inactivation method, followed by molecular analysis to identify genes with the Streck ARM-D kit, β-Lactamase assay.

### Case definition

A case was defined as a patient with *Escherichia coli, Enterobacter cloacae* complex, or *Klebsiella* species resistant to one or more carbapenems, isolated from urine or normally sterile specimens collected from residents within a designated surveillance area. This surveillance area comprised eight counties surrounding Nashville, Tennessee. The first occurrence of each organism that met the above case definition in a 30-day period was eligible for inclusion as an incident case in this evaluation (referred to as the incident case).

CRE cases were evaluated to ascertain whether the infections were associated with healthcare exposure or community-associated. Healthcare exposure was assessed based on a specific EIP protocol and was not directly tied to the National Healthcare Safety Network definitions of healthcare-associated infections. Patients were considered to have healthcare exposure if they had been admitted to an acute care hospital, a long-term care facility, or a nursing home, or if they had undergone surgery within the year prior to specimen collection. Additionally, patients were classified as having healthcare exposure if they had an indwelling device at the time of specimen collection or within two calendar days before it, or if they were current chronic dialysis patients.

### Statistical analysis

Community- and healthcare-associated infections were examined using the chi-square test. For continuous variables that adhered to a normal distribution, an independent Student *t*-test was conducted, whereas the Wilcoxon Rank Sum test was used for continuous variables that did not conform to a normal distribution. A multivariable logistic regression analysis was performed to describe significant predictors of clinical outcomes. All variables relevant to clinical outcomes, based on theoretical reasoning and review of previous studies, were included in the model. Race was not included as it did not have a significant association with the outcome variables in these data and did not improve the model’s fitness. A two-tailed *p*-value of <0.05 was considered statistically significant. Data analysis was performed using SAS version 9.4.

## Results

### CRE trends and CP-CRE characteristics

**Figure 1.**
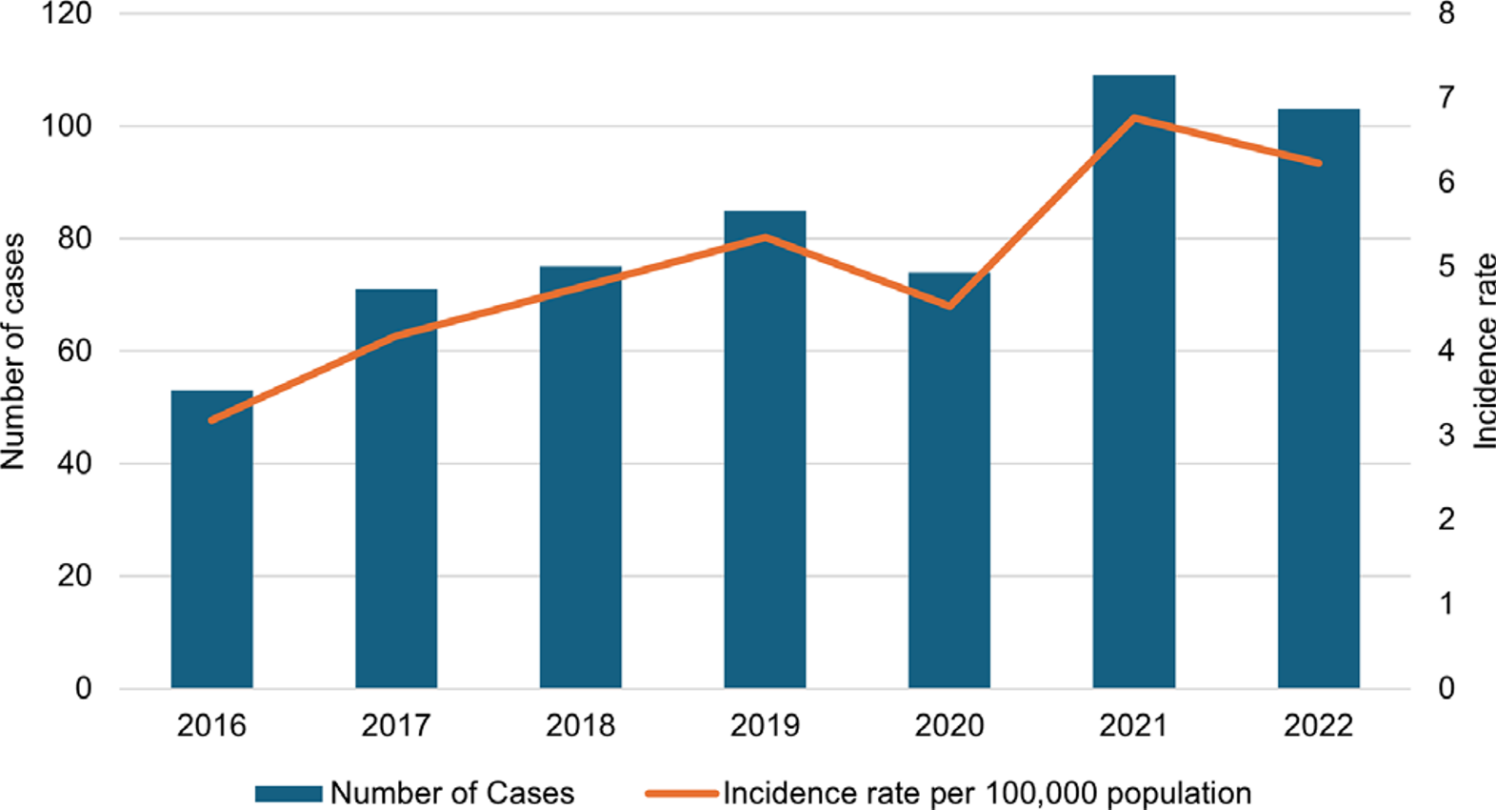
CRE trends and incidence rate per 100 000 population. The solid orange line represents the incidence rate. CRE, carbapenem-resistant Enterobacterales.

Between 2016 and 2022, 570 CRE cases of CRE were reported (Figure 1). The incidence rate increased from 3.18 cases per 100 000 population in 2016 to 6.23 cases per 100 000 population in 2022 (*p* ≤ 0.0001).

**Figure 2.**
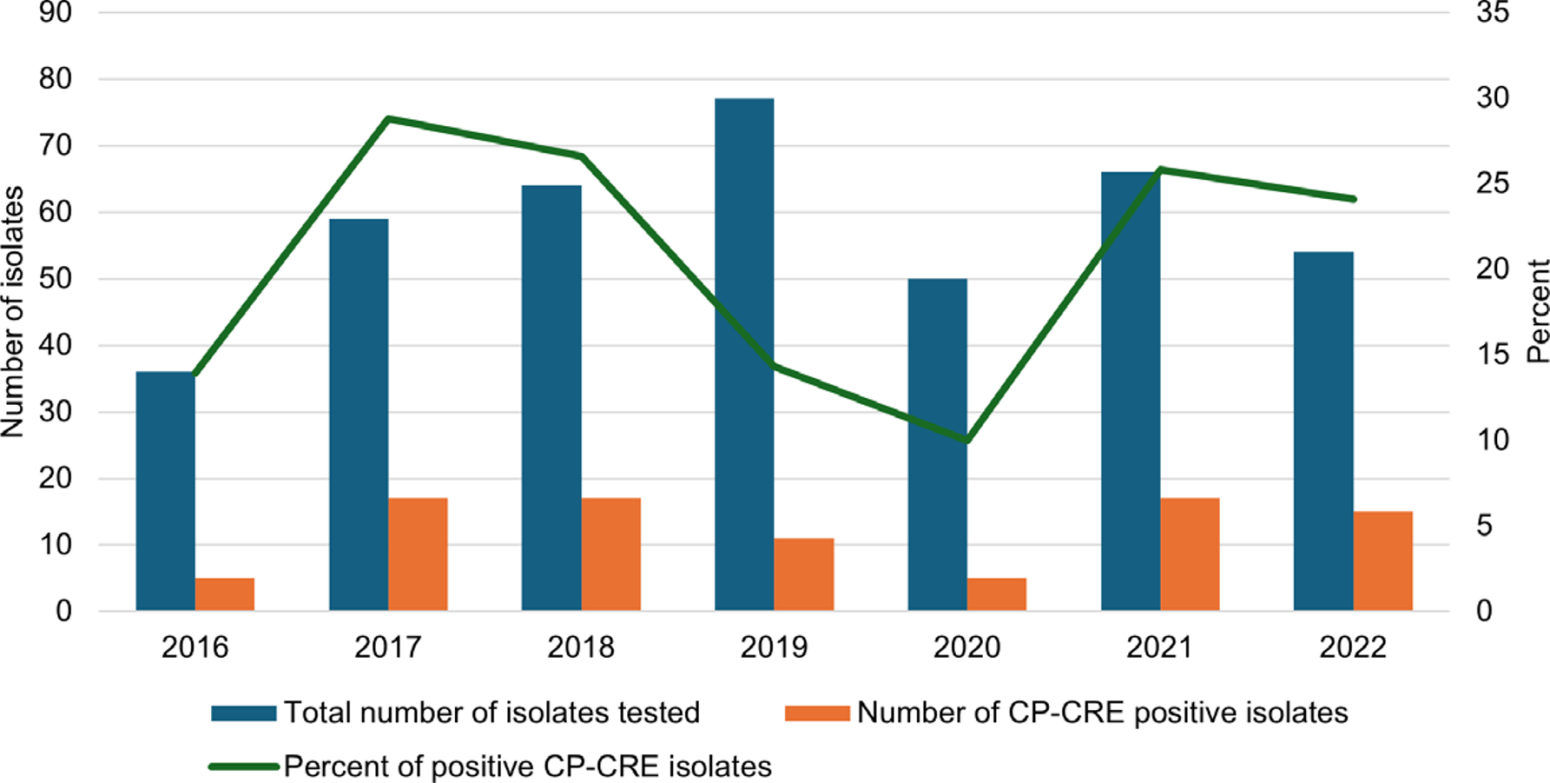
Trends of CRE and CP-CRE. The blue bars indicate the number of CRE isolates tested for carbapenemase production. The orange bars represent the number of CRE isolates that tested positive for carbapenemase production. The solid green line shows the trend in the percentage of carbapenemase-positive cases. CP-CRE, carbapenemase-producing carbapenem-resistant Enterobacterales; CRE, carbapenem-resistant Enterobacterales.

In accordance with the study protocol, CRE isolates were sent from the originating clinical laboratories to the Tennessee State Public Health Laboratory for carbapenemase testing. A total of 406 (71.2%) isolates were analysed, and 87 (21.4%) were determined to be CP-CRE (Figure 2). In 2016, there were five CP-CRE isolates, which increased to 17 in both 2017 and 2018. The number fell to 11 in 2019 and decreased to 5 in 2020, before rising again to 17 in 2021 and dropping to 15 in 2022.

The positivity rate, which indicates the percentage of tested isolates that were positive for carbapenemase production, was 13.9% in 2016 (Figure 2). This figure rose to 28.8% in 2017, followed by a slight decrease to 26.6% in 2018. However, it dropped to 14.3% in 2019 and declined to 10% in 2020. In the subsequent years, the positivity rate increased again, reaching 25.8% in 2021 and 24.1% in 2022.

**Figure 3.**
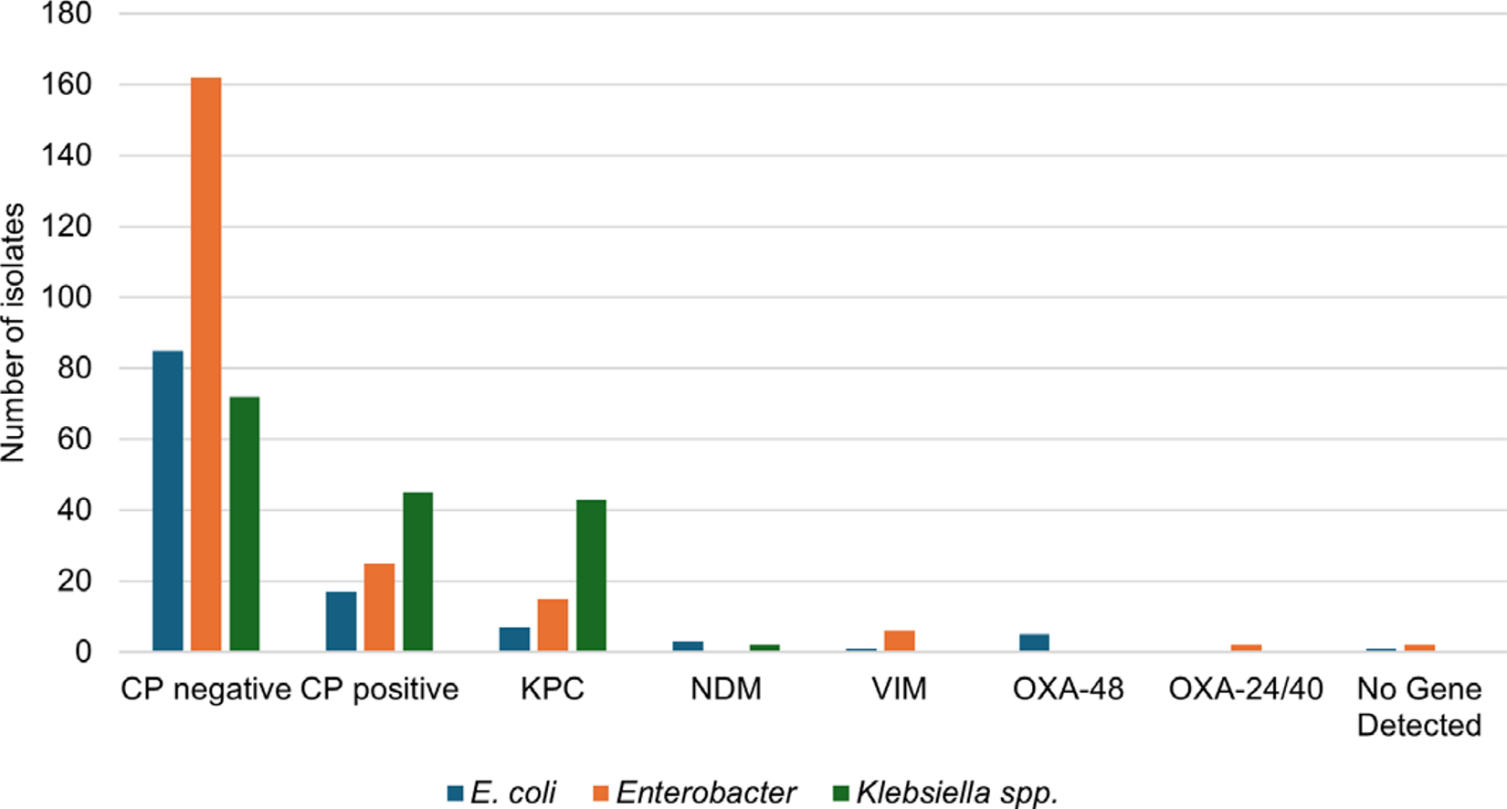
Carbapenem-resistant and carbapenemase-producing organisms and genes. A subset of CRE isolates was tested for carbapenemase production. This figure demonstrates the total number of isolates negative for carbapenemase (CP) production (far left) and positive for CP production (second from left). This figure also depicts the number of *E. coli* (blue bars), *Enterobacter* (orange bars), and *Klebsiella* spp. (green bars) that were positive for KPC, NDM, VIM, or OXA carbapenemase. CP-CRE = carbapenemase-producing carbapenem-resistant Enterobacterales; CRE = carbapenem-resistant Enterobacterales; KPC = *Klebsiella pneumoniae* carbapenemase; NDM = New Delhi metallo-β-lactamase; OXA = Oxacillinase; VIM = Verona integron-encoded metallo-β-lactamase.

The isolates submitted for carbapenemase testing were analysed to identify the organisms responsible for the infections and to determine the specific genes associated with carbapenemase production (Figure 3). Among the tested isolates, *Enterobacter* species were the most frequently identified, accounting for 247 cases (43.3%). Of these *Enterobacter* isolates, 162 tested negative for carbapenemase, whereas 25 tested positive. *Klebsiella* species comprised 117 isolates (28.8%); within this group, 45 tested positive for carbapenemase and 72 tested negative. *E. coli* isolates accounted for 102 cases (25.1%); 85 isolates were negative for carbapenemase, whereas 17 were positive.

The highest rate of carbapenemase production was observed in *Klebsiella* species, with 72 positive isolates, corresponding to 38.1% of the total samples analysed (Figure 3). The predominant carbapenemase-producing gene detected in this study was *Klebsiella pneumoniae* carbapenemase (KPC), which was found in 65 isolates, representing 74.7% of the tested isolates. *Klebsiella* species were the most prevalent among the KPC-positive organisms, accounting for 43 isolates or 66.2% of the total KPC-positive findings. Additionally, several less prevalent genes were identified, including New Delhi metallo-β-lactamase (NDM) (*N* = 5; 5.7%), Verona integron-encoded metallo-β-lactamase (VIM) (*N* = 7; 8.0%), Oxacillinase (OXA)-48 (*N* = 5; 7.0%), and OXA24/40 (*n* = 2; 2.3%). Three isolates tested negative for all targets, and no additional testing was conducted on these isolates.

### Patient characteristics

Demographics, admission status, and mortality rates of patients were analysed to identify any significant differences among these variables (Table 1). The median age of the patient cohort was 69.5 years. In this study, 269 patients (66.3%) were female, and 137 patients (33.7%) were male. The mean incidence rate of CRE was 5.1 per 100 000 and 4.8 per 100 000 population among Blacks and Whites, respectively (*p* = 0.69; results not shown). Out of the total cases, 318 (78.3%) were White, 60 (14.8%) were Black, 4 (1.0%) were of other races, and the race was unknown for 24 cases (5.9%). There was no significant difference in the racial distribution between CP-CRE and non-CP-CRE cases. Overall, 47.5% of patients were admitted to a hospital. A higher proportion of CP-CRE cases (60.9%) required hospital admission compared to non-CP-CRE cases (43.9%) (*p* = 0.004). Among those tested for carbapenemase, 17.2% of patients infected with CP-CRE were admitted to the intensive care unit (ICU) compared to 10.7% of non-CP-CRE cases (*p* = 0.093). The median length of hospital stay was 7 days for patients infected with CP-CRE and 6 days for patients with non-CP-CRE (*p* = 0.392). The overall 30- and 90-day mortality rates for patients infected with CRE were 7.4% and 14.7%, respectively. A total of 5.7% of cases infected with CP-CRE died within 30 days of specimen collection, compared to 3.5% of cases infected with non-CP-CRE (*p* = 0.323). However, within 90 days of specimen collection, 20.7% of CP-CRE cases and 9.1% of non-CP-CRE cases resulted in death (*p* = 0.003).

**Table 1.** Demographic and hospital admission of patients infected with CRE

Variable	All CRE N = 406	CP-CRE N = 87	Non-CP-CRE N = 319	p-Value
Median age	69.5	67	70	0.11
Sex				
Female	269 (66.3%)	52 (59.8%)	217 (68%)	0.15
Male	137 (33.7%)	35 (40.2%)	102 (32%)	0.14
Race				
Black	60 (14.8%)	16 (18.4%)	44 (13.8%)	0.284
White	318 (78.3%)	64 (73.6%)	254 (79.6%)	0.224
All other races^a^	4 (1%)	2 (2.3%)	2 (0.6%)	0.162
Unknown race	24 (5.9%)	5 (5.7%)	19 (6%)	0.942
Hospital admission status				
Hospital admission	193 (47.5%)	53 (60.9%)	140 (43.9%)	**0.004**
ICU admission	49 (12.1%)	15 (17.2%)	34 (10.7%)	0.093
Median length of hospital stay (days)	6	7	6	0.392
Mortality				
30-day mortality^b^	30 (7.4%)	5 (5.7%)	11 (3.5%)	0.323
90-day mortality^b^	47 (14.7%)	18 (20.7%)	29 (9.1%)	**0.003**

This table reports the median age, sex, race, hospital admission, and 30- and 90-day mortality of all patients with CRE, non-CP-CRE (isolates negative for carbapenemase production), and CP-CRE (isolates positive for carbapenemase production). The values in this table are represented as numbers (*N*) and percentages (%). *P*-values compare patients with non-CP CRE to CP-CRE isolates, and *p*-values of ≤0.05 are considered significant. Abbreviations: CP-CRE, carbapenemase-producing carbapenem-resistant Enterobacterales; CRE, carbapenem-resistant Enterobacterales.

The bold entries signifies that the value is less than 0.05 indicating statistically significant association.

a Asian, Native Hawaiian or Pacific Islander, American Indian/Alaska Native.

b Death rate within 30 or 90 days of specimen collection.

**Figure 4.**
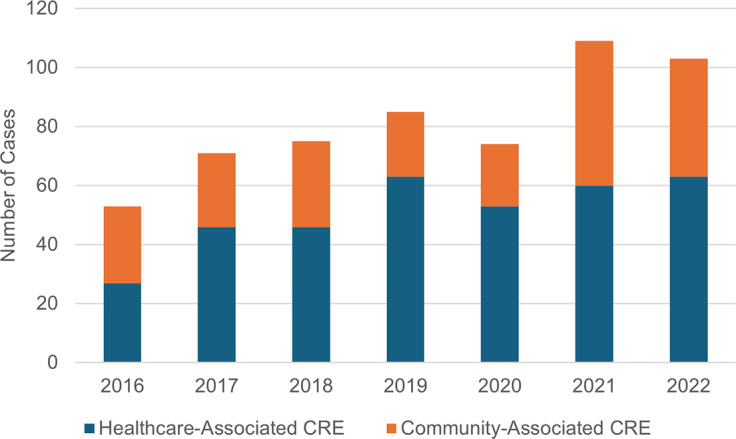
Healthcare-associated and community-associated CRE infections. Cases were evaluated to determine whether they were associated with a previous healthcare exposure (blue) or the community (orange). CRE = carbapenem-resistant Enterobacterales.

Among the 570 CRE cases identified in the study, 358 (62.8%) were categorized as healthcare-associated, whereas 212 (37.2%) were classified as community-associated (Figure 4). Both community-associated and healthcare-associated CRE cases increased in frequency between 2021 and 2022. No statistically significant change was observed in the proportion of community-associated CRE over the evaluated years (*p* = 0.94). Among the 87 isolates positive for carbapenemase production, 71 (81.6%) isolates were identified as healthcare-associated (data not shown). There were very few community-associated CP-CRE cases during the surveillance period, and the overall trend did not change during this time (data not shown).

The most prevalent comorbidity identified among patients was diabetes mellitus, which was observed in 210 patients (36.8%) with CRE. This was followed by cerebrovascular accidents, solid tumours, and congestive heart failure, which were reported in 101 (17.7%), 100 (17.5%), and 91 (15.9%) of all CRE patients, respectively (Table 2).

**Table 2.** Common co-morbidities of patients with CREs, non-CP-CRE, and CP-CRE

	All CREs (N)	Non-CP-CRE (N)	CP CRE (N)
AIDS	1	0	0
Cerebrovascular accidents	101	53	26
Chronic liver disease	13	6	5
Chronic renal disease	49	33	10
Congestive heart failure	91	49	16
Connective tissue disease	10	5	1
Dementia	70	43	7
Diabetes	210	116	38
Diabetes with chronic complications	50	26	8
Hematologic malignancy	11	4	3
Hemiplegia	22	9	7
Metastatic tumour	24	15	4
Myocardial infarction	47	24	12
Peptic ulcer disease	15	9	3
Peripheral vascular disease	73	36	19
Solid tumour	100	46	24

Abbreviations: CP-CRE, carbapenemase-producing carbapenem-resistant Enterobacterales; CRE, carbapenem-resistant Enterobacterales.

### Multivariable regression models

A logistic regression model was developed to analyse the 30- and 90-day outcomes following the specimen collection date. This model accounted for carbapenemase production status, race, age, sex, and the CCI score (Table 3). Age and race were not associated with this analysis’s 30- or 90-day mortality rate. The CCI score was found to have a significant association with 30-day mortality, yielding an adjusted odds ratio (OR) of 1.28 (95% CI, 1.10–1.49; *p* ≤ 0.0014). Conversely, patients with CP-CRE did not demonstrate a significant correlation with 30-day mortality, as reflected by an adjusted OR of 1.69 (95% CI, 0.73–3.91; *p* = 0.2194). Patients infected with CP-CRE did, however, exhibit a substantially elevated risk of 90-day mortality. Specifically, the adjusted odds of mortality for these patients were 2.22 times greater (OR, 2.22; 95% CI, 1.12–4.42; *p* ≤ 0.0001) compared to those infected with non-CP-CRE. Furthermore, the results indicated that for every one-point increase in the CCI score, the adjusted odds of death increased by 1.33 (OR, 1.33; 95% CI, 1.16–1.52; *p* ≤ 0.0001). The 90-day mortality ratio among females was 0.57 compared to males, although this difference was not statistically significant (OR, 0.57; 95% CI, 0.29–1.09; *p* = 0.0919). The adjusted OR for patients infected with CP-CRE admitted to the hospital within 30 days of specimen collection was 1.77 (95% CI, 1.06–2.98; *p* = 0.03) compared to patients without CP-CRE infection. Additionally, the adjusted OR for female patients admitted to the hospital within 30 days of specimen collection was determined to be 0.70 relative to males (OR, 0.70; 95% CI, 0.45–1.09; *p* = 0.11). The adjusted OR for patients infected with CP-CRE admitted to the ICU within 7 days of specimen collection was 1.40 compared to non-CP-CRE patients (95% CI, 0.703–2.77; *p* = 0.34). Furthermore, the adjusted OR for female patients admitted to the ICU within 7 days of specimen collection was 0.58 compared to males (95% CI, 0.311–1.078; *p* = 0.09). As anticipated, the CCI scores demonstrated a significant correlation with both hospital and ICU admissions (OR, 1.24; 95% CI, 1.11–1.37; *p* < 0.001; and OR, 1.19; 95% CI, 1.05–1.36; *p* = 0.01, respectively).

**Table 3.** Multivariate models of CRE and non-CP-CRE patients

Characteristic	Adjusted odds ratio (95% Cl)	P-value
30-day mortality		
CP-CRE	1.69 (0.73–3.91)	0.2194
Female	0.99 (0.44–2.21)	0.0919
Age	1.02 (0.99–1.05)	0.2492
CCI score	1.28 (1.10–1.49)	**<0.0014**
90-day mortality		
CP-CRE	2.22 (1.12–4.42)	**<0.0001**
Female	0.57 (0.29–1.09)	0.0919
Age	1.01 (0.99–1.03)	0.283
CCI score	1.33 (1.16–1.52)	**<0.0001**
Hospital admission		
CP-CRE	1.77 (1.06–2.98)	**0.03**
Female	0.70 (0.45–1.09)	0.11
Age	1.04 (0.99–1.02)	0.47
CCI score	1.24 (1.11–1.37)	**<0.0001**
ICU admission		
CP-CRE	1.4 (0.70–2.77)	0.34
Female	0.58 (0.31–1.08)	0.09
Age	0.985 (0.97–1.00)	0.09
CCI score	1.19 (1.05–1.36)	**0.01**

To determine odds ratios, multivariate models of patients with CP-CRE (*N* = 87) and non-CP-CRE (*N* = 319) were evaluated. Abbreviations: CCI, Charlson Comorbidity Index; CI, confidence interval; CP-CRE, carbapenemase-producing carbapenem-resistant Enterobacterales; CRE, carbapenem-resistant Enterobacterales; ICU, intensive care unit.

The bold entries signifies that the value is less than 0.05 indicating statistically significant association.

## Discussion

We observed a significant increase in cases of CRE and CP-CRE in Tennessee between 2016 and 2022. These findings align with other studies indicating similar trends across the United States, including Houston, during the same period [8, 9]. However, our results contradict a previous publication that reported a decline in CRE and CP-CRE cases in the United States from 2016 to 2020 [10]. This discrepancy may be attributed to geographic variations, as our analysis focused specifically on Tennessee, whereas the earlier study included data from ten states, including Tennessee.

Throughout the study period, we noted an upward trend in CP-CRE incidence. Carbapenemase positivity doubled in 2021 and 2022 compared to 2020. The CDC supported these findings in its Antimicrobial Resistance Threat Report published in July 2024, attributing the considerable rise post-2020 to disruptions in infection control practices within healthcare facilities caused by the COVID-19 pandemic [2].

Among the pathogens identified, *Enterobacter* species emerged as the predominant organism, with *Klebsiella* species being the most prevalent among CP-CRE isolates. This aligns with other epidemiological studies that similarly recognized *Klebsiella* species as the most frequently reported CP-CRE [11–14]. We found KPC to be the most common carbapenemase-producing gene, accounting for 74.7% of identified carbapenemase genes, followed by VIM, OXA, and NDM. Other studies have corroborated KPC’s status as the leading gene mediating carbapenem resistance in the United States [8, 9, 15]. Infections caused by KPC-producing *Klebsiella* species are associated with a heightened risk of bloodstream infections, which can result in severe illness [16]. Interestingly, while KPC was prevalent among *Klebsiella* species, VIM was more frequently found among *Enterobacter* species. This variance may be due to the geographic scope of our study population. Understanding the local epidemiology of CRE pathogens and their resistance mechanisms is essential for the early identification and effective treatment of infections that could lead to serious complications. While the overall prevalence of carbapenemase genes may fluctuate over time, continuous monitoring of these trends is crucial to deepen our understanding of the epidemiology of CP-CRE.

In this study, we assessed the characteristics of patients with CRE and compared those with CP-CRE and those with non-CP-CRE. Most patients with CRE were older, particularly those aged 60 and above. Our findings revealed that individuals 80 and older were more likely to be infected with non-CP-CRE. The reasons behind this observation are not yet clear and require further investigation to uncover the contributing factors. We also noted that the average incidence of CRE infections was slightly higher among Black patients; however, this difference was not statistically significant. Previous studies have suggested that race is not a significant factor in developing CRE infections [12]. Regarding mortality rates, we found that 7.4% of patients died within 30 days and 16.2% within 90 days after specimen collection. These figures are significantly lower than the 60% mortality rate reported for cases of CRE bacteraemia [17]. In the literature, the mortality rates associated with CRE vary significantly [6, 18–20]. These differences may be related to the types of infections and the specific pathogens responsible for those infections. This variability highlights the need for further analysis to determine whether the infection type and organism influence the observed differences in mortality rates. Moreover, our study indicated that 62.8% of CRE cases were linked to healthcare exposure, reinforcing earlier research highlighting healthcare settings as a significant risk factor for CRE infections [12, 21, 22].

Our observations indicate that patients with CRE infections exhibit chronic illnesses such as diabetes mellitus, cerebrovascular accidents, congestive heart failure, and solid tumours. These conditions predominantly affect older populations and necessitate frequent visits to healthcare facilities, thereby increasing the risk of exposure to infections caused by drug-resistant pathogens. Overall, these findings emphasize the necessity for ongoing research to better understand the dynamics of CRE infections and the factors influencing patient outcomes.

Our analysis reveals a significant correlation between 30-day mortality rates and the CCI score. This finding underscores the CCI score as the most reliable predictor of severe clinical outcomes among patients infected with CRE, a point supported by existing literature [23]. Interestingly, our study found no significant difference in 30-day mortality rates between infections caused by CP-CRE and those caused by non-CP-CRE organisms. This observation aligns with a previous study [24]. However, we identified a significant association between the 90-day mortality rates, the CCI score, and the carbapenemase production status of the infecting pathogens. After accounting for various factors, we found no significant relationship between age and 30- or 90-day mortality rates. This suggests that a more thorough investigation may be necessary to fully understand age’s impact on clinical outcomes. Finally, our analysis indicated no statistically significant difference in mortality rates between male and female patients once we adjusted for the CCI score, age, and carbapenemase production status.

A thorough understanding of the epidemiology and risk factors related to the clinical outcomes of patients infected with CRE and CP-CRE is essential. This knowledge enables early case identification, helps mitigate the spread of infection, and improves clinical outcomes. While our study has limitations, including a restricted surveillance area and the fact that we did not analyse the relationship between specific organisms and mortality, it provides a framework for better understanding the characteristics of CRE in Tennessee.

Patients with CP-CRE demonstrate a significantly elevated risk of mortality within 90 days following specimen collection compared to those infected with non-CP-CRE strains. Therefore, it is imperative to use carbapenemase test results for surveillance and to inform appropriate treatment strategies, ensuring timely infection prevention measures. In our surveillance area, only one clinical lab tests isolates for carbapenemase production or the presence of the gene responsible for carbapenemase production. All other labs within the surveillance area send their isolates to the Tennessee State Public Health Lab for carbapenemase testing, primarily for surveillance purposes. This study indicated that infection with CP-CRE is independently and significantly associated with poor clinical outcomes in patients. Other studies showed that early appropriate antibiotic administration in treating CP-CRE cases is associated with improved clinical outcomes [17]. In addition, adjusting the patient treatment based on the types of carbapenemase genes identified is also essential, as effective antimicrobial therapy may vary by gene and organism combination [16]. Given that CRE and CP-CRE continue to present considerable public health challenges, laboratories should consider enhancing their testing capabilities to facilitate the timely detection of CP-CRE. Additionally, effectively using available testing resources at public health laboratories is crucial for improving patient care and infection control. It is vital to monitor emerging trends and contemplate expanding infection control efforts beyond the confines of hospitals and skilled nursing facilities.

## Data Availability

The data presented are not publicly accessible due to the confidentiality of protected health information. All inquiries regarding the data may be directed to the corresponding author (D.M.), who will evaluate data requests in accordance with the data release policy established by the Tennessee Department of Health.
